# Supplementary material for: Multi-layered proteomic analyses decode compositional and functional effects of cancer mutations on kinase complexes
Source: Nat Commun. 2020 Jul 16;11:3563. doi: 10.1038/s41467-020-17387-y (PMC7366679; doi:10.1038/s41467-020-17387-y)
Supplement: Supplementary file 1 — Supplementary Information [file 41467_2020_17387_MOESM1_ESM.pdf]

# - Supplementary Information-

Multi-layered proteomic analyses decode compositional and functional effects of cancer mutations on kinase complexes

Martin Mehnert[1*], Rodolfo Ciuffa[1], Fabian Frommelt[1], Federico Uliana[1], Audrey van Drogen[1], Kilian Ruminski[1,3], Matthias Gstaiger[1*] and Ruedi Aebersold[1, 2*]

1. *Department of Biology, Institute of Molecular Systems Biology, ETH Zurich, Switzerland*
2. *Faculty of Science, University of Zurich, Switzerland*
3. *Current address : Centre d'Immunologie de Marseille-Luminy, Aix Marseille Université, INSERM, CNRS, 13288 Marseille, France*

*Corresponding authors:
aebersold@imsb.biol.ethz.ch; gstaiger@imsb.biol.ethz.ch; mehnert@imsb.biol.ethz.ch

Supplementary Figure 1

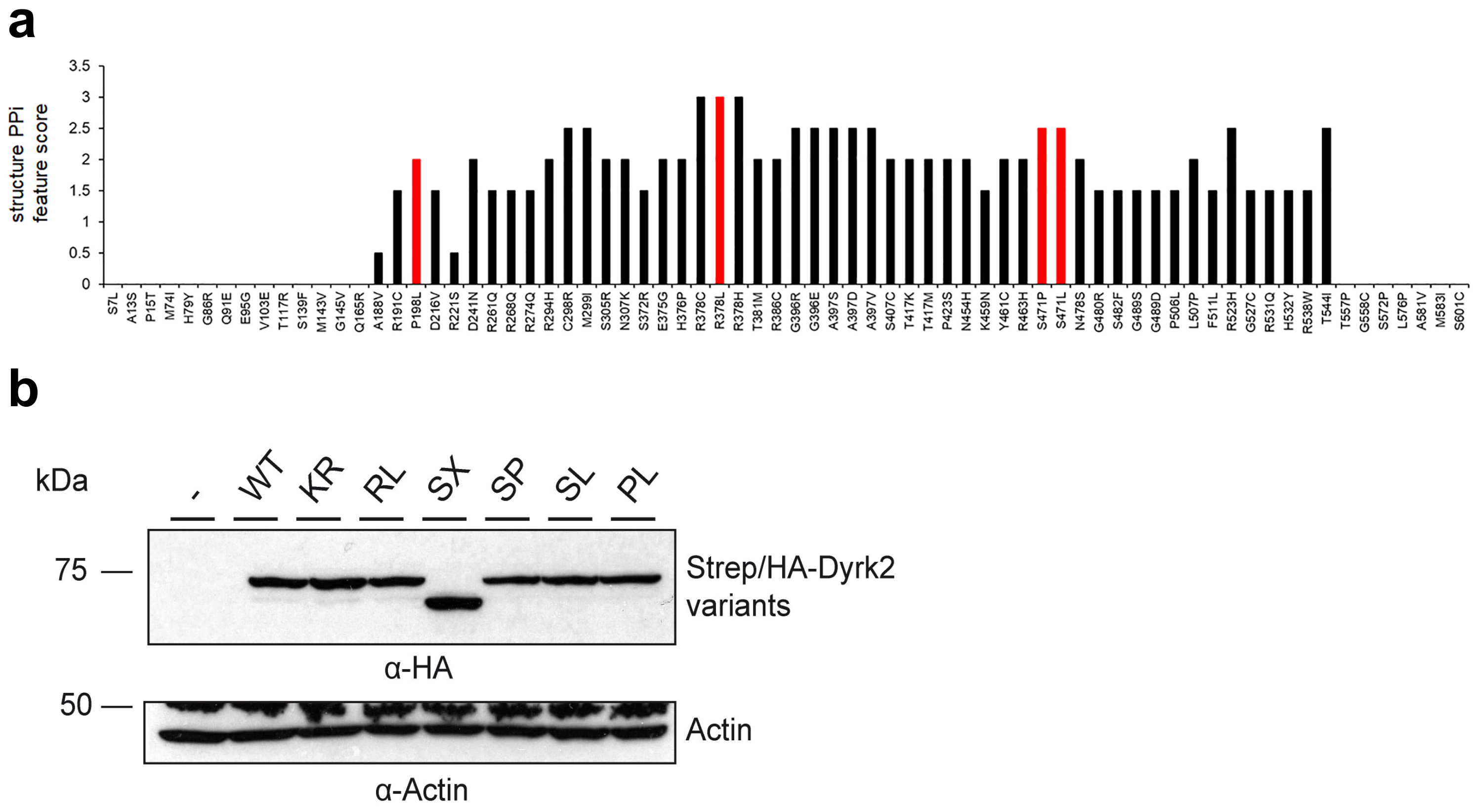

Supplementary Figure 1. Selection and expression of cancer-related Dyrk2 point mutants. (**a**) Distribution of the structure ppi damage probability score across all Dyrk2 missense mutations (n = 72) annotated in the COSMIC database (11.08.2015). The damage probability score of Dyrk2 mutations selected for this study is highlighted in red. (**b**) Expression level of Strep/HA-tagged Dyrk2 mutant variants in T-REx HEK293 Flp-in cells determined by western blot analysis. The expression of the Dyrk2 constructs was induced by addition of doxycycline. The experiment was repeated independently (n = 2) with similar results. Source data are provided as source data file.

Supplementary Figure 2

a

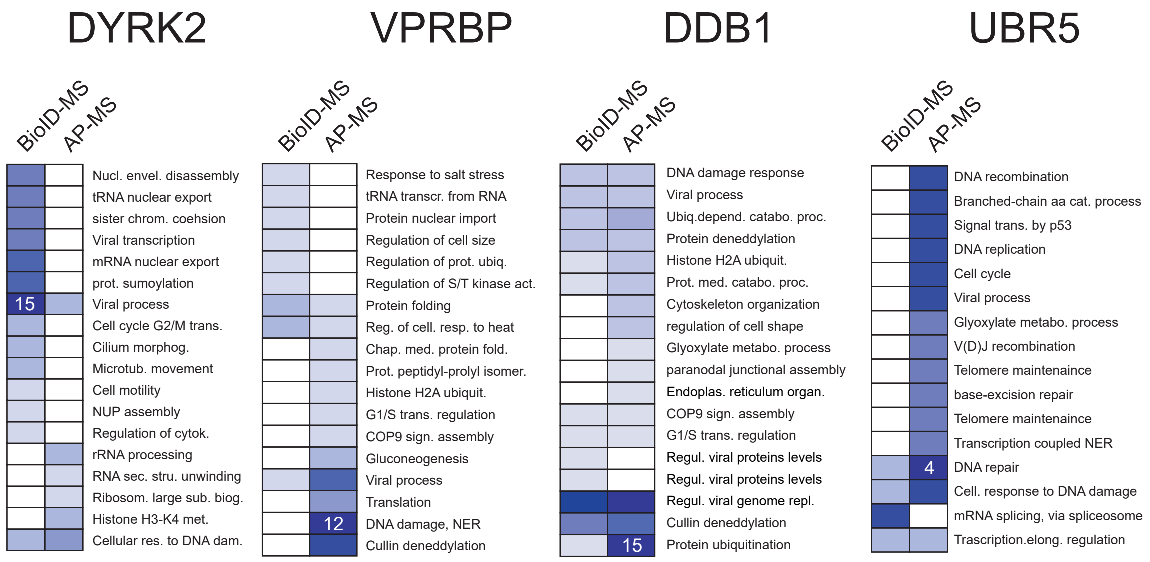

Supplementary Figure 2. Interaction network analysis of the Dyrk2-EDVP complex. (**a**) Biological processes GO terms of interactors found for the individual Dyrk2-EDVP complex components. The color intensity indicates the number of interactors assigned with the respective GO term.

Supplementary Figure 3

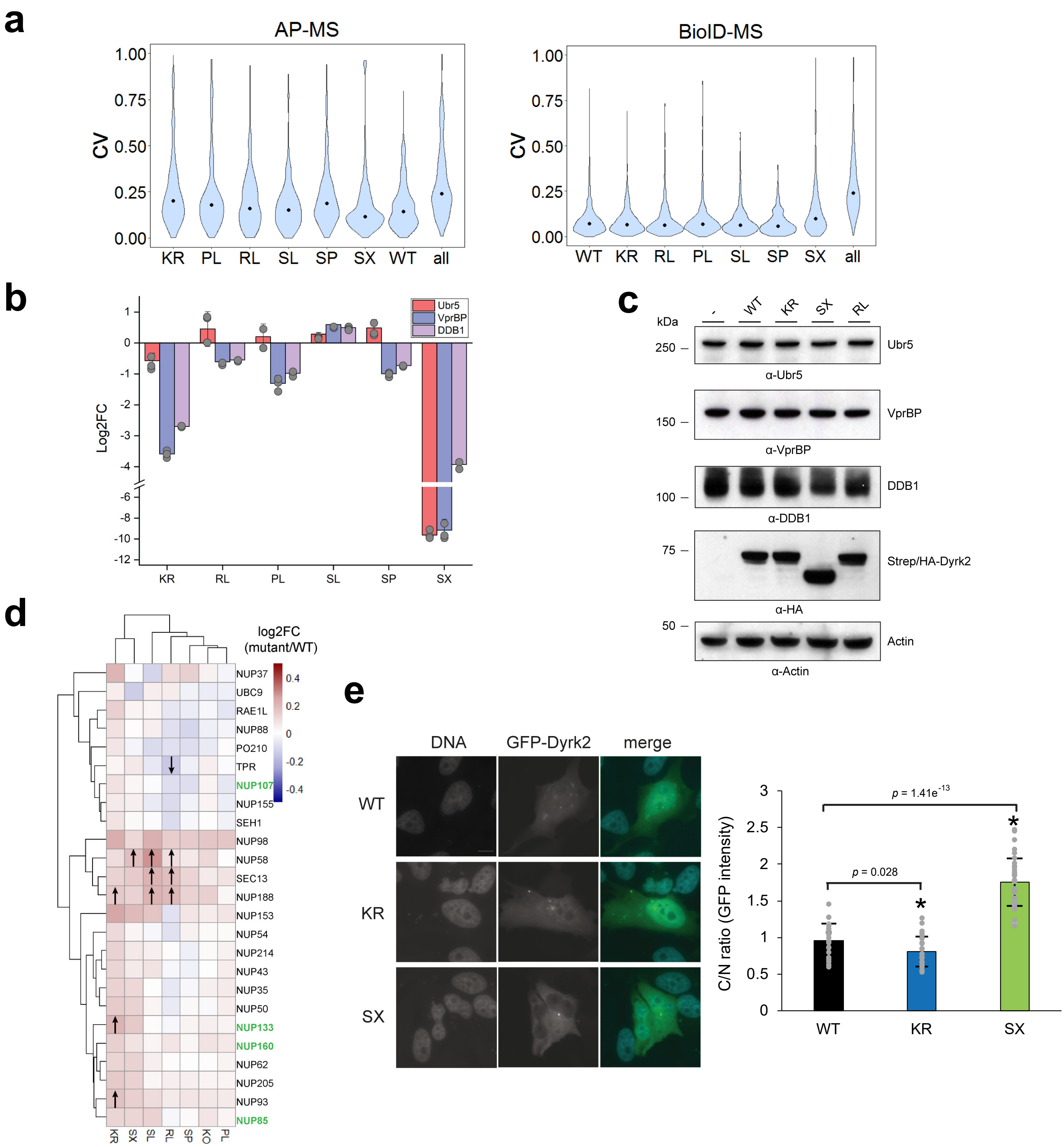

Supplementary Figure 3. Differential interaction analysis of Dyrk2 point mutants by AP-MS and BioID-MS. (**a**) Peptide CV distribution for triplicates of the different Dyrk2 mutants from AP-MS (left panel) and BioID-MS (right panel) experiments. (**b**) Effect of cancer related mutations on the interaction to the core subunits of the Dyrk2-EDVP complex measured by BioID-MS. Error bars represent the mean value with 95% confidence interval (n = 3 biologically independent experiments). (**c**) Expression level of the EDVP complex components in the different Dyrk2 mutant cell lines determined by western blot analysis. The experiment was repeated independently (n = 2) with similar results. (**d**) Heatmap illustrating changes in the protein abundances of nuclear pore proteins identified in this study. The arrow indicates the direction of a significant change in protein abundance (adj. p-value ≤ 0.05). Members of the nuclear Y-complex are highlighted in green letters. The statistical analysis and p-value calculation (adj. p-value (FDR)) was performed within the mapDIA package. (**e**) Cellular localization of GFP-tagged wt and mutant Dyrk2 determined by immunofluorescence microscopy. Representative immunofluorescence images of T-REx HeLa cells transfected with different Dyrk2 variants (left panel). Ratio of the GFP intensity measured in the nucleus (N) and the cytoplasm (C), respectively (right panel) (WT (n = 22 cells), KR (n = 24 cells), SX (n = 37 cells)). Error bars represent the standard deviation of the mean value (centre). Statistical analysis was performed with two-tailed unpaired Student's t-test. The asterisk indicates a significant statisitical difference (p-value ≤ 0.05). Scale bar = 10 μm. Source data are provided as source data file.

Supplementary Figure 4

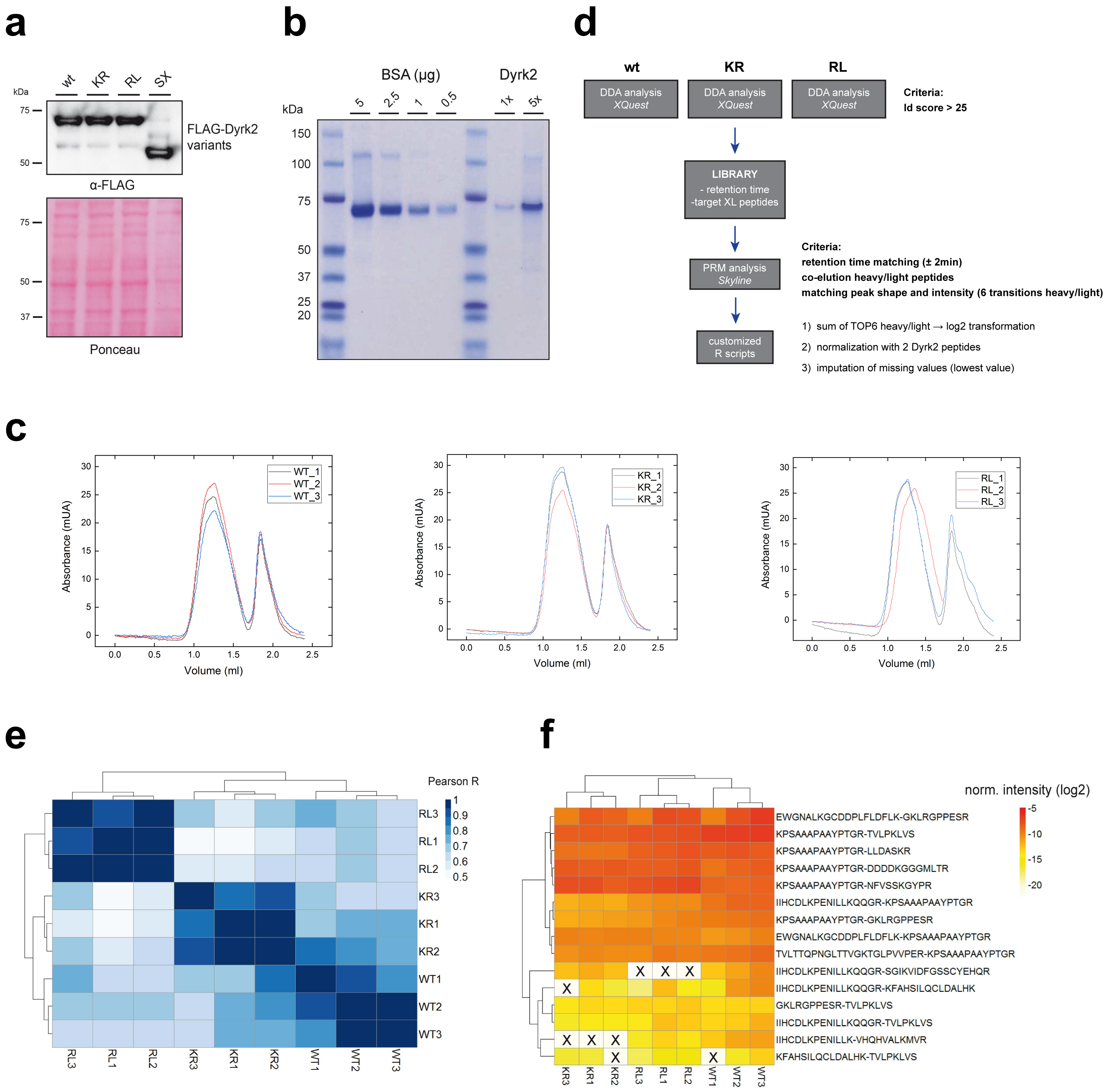

Supplementary Figure 4. Purification of Dyrk2 mutants from SF9 insect cells and targeted cross-linking MS of the purified kinase variants. (**a**) Recombinant expression of FLAG-tagged Dyrk2 mutants in SF9 insect cells determined by western blot analysis. The experiment was repeated independently (n = 3) with similar results. (**b**) SDS-PAGE of FLAG-Dyrk2 purified from SF9 insect cells after gelfiltration and concentration of the elution fractions. The purified Dyrk2 sample was subjected to crosslinking with DSS. The experiment was repeated independently with similar results (n = 3). (**c**) Chromatographic peptide-SEC profile of digested crosslinked proteins from purified Dyrk2 mutants and their replicates. For the following analysis of crosslinked peptides by mass spectrometry fractions between 1.0 and 1.3 ml elution volume were collected. (**d**) Scheme of the workflow for the differential cross-linking MS data analysis of the Dyrk2 mutants. Crosslinked peptides of the different mutant conditions were first identified by the software XQuest using a Id score threshold of ≥ 25 followed by a targeted quantification of the identified crosslinked peptides by PRM. To be selected for quantification by PRM following criteria have to be fulfilled: 1) the crosslinked peptide elutes in a retention time window of ± 2 min across the different conditions; 2) co-elution of the heavy and the light form of the cross-linked peptide; 3) matching of peak shape and intensity for six common transitions of heavy and light cross-linked peptides. (**e**) Correlation of the log2 transformed transition intensity (top5) normalized to non-cross linked Dyrk2 peptides of the different Dyrk2 mutant conditions and their replicates. (**f**) Hierarchical clustering of the nine crosslinking samples using normalized transition intensity of crosslinked peptides as in (**e**). Source data are provided as source data file.

Supplementary Figure 5

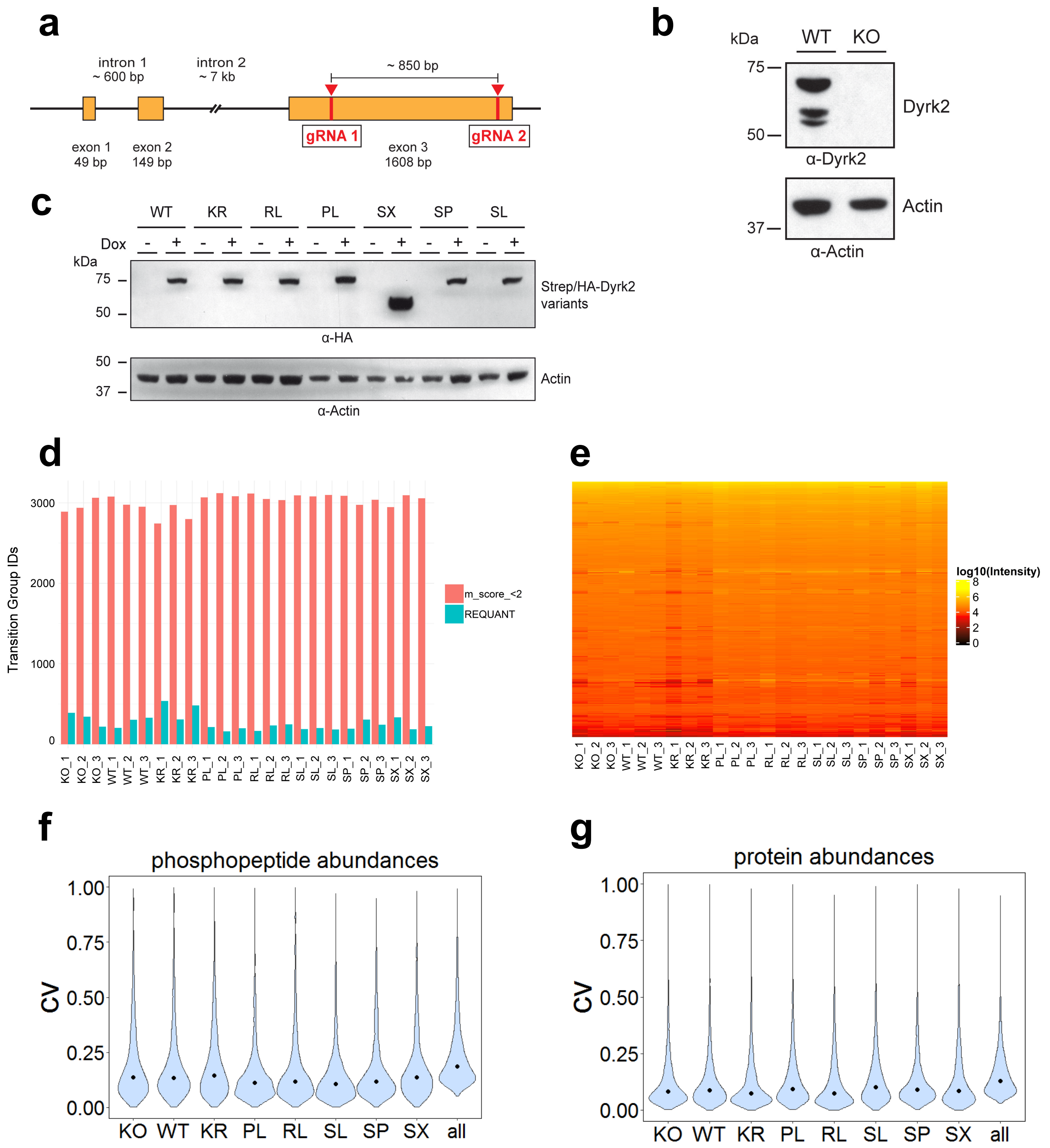

Supplementary Figure 5. Global proteomic and phosphoproteomic analysis of the cancer-related Dyrk2 mutants. (**a**) Scheme of the gRNA targeting strategy to generate a Dyrk2 KO cell line by CRISPR/Cas9. For the gene deletion a gRNA pair (gRNA1/2) targeting the third exon was used. (**b**) Expression analysis of Dyrk2 in wildtype and CRISPR/Cas9 engineered Dyrk2 KO T-REx HeLa cells. The experiment was repeated independently (n = 3) with similar results. (**c**) Expression level of the different Dyrk2 variants genomically integrated into T-REx HeLa Dyrk2 KO cells. The expression of the Dyrk2 constructs was induced by the addition of doxycycline (Dox). The experiment was repeated independently (n = 2) with similar results. (**d**) Distribution of the number of phosphopeptides in each sample detected by DIA using openSWATH and a phospho-specific DDA library created with LuciPHOr2. Red bars represent phosphopeptides identified with a m-score < 2 whereas blue bars show the number of phosphopeptides identified by the Requant function of openSWATH (m-score > 2). (**e**) Hierarchical clustering of log10 transformed phosphopeptide intensity across different mutant conditions. (**f**) CV distribution of phosphopeptide intensity for sample replicates and all samples combined. The replicate CV is below 16 % in each sample replicate. (**g**) CV distribution of protein intensity of the total proteome analysis for sample replicates and all samples combined. The replicate CV is below 11 % in each sample replicate. Source data are provided as source data file.

Supplementary Figure 6

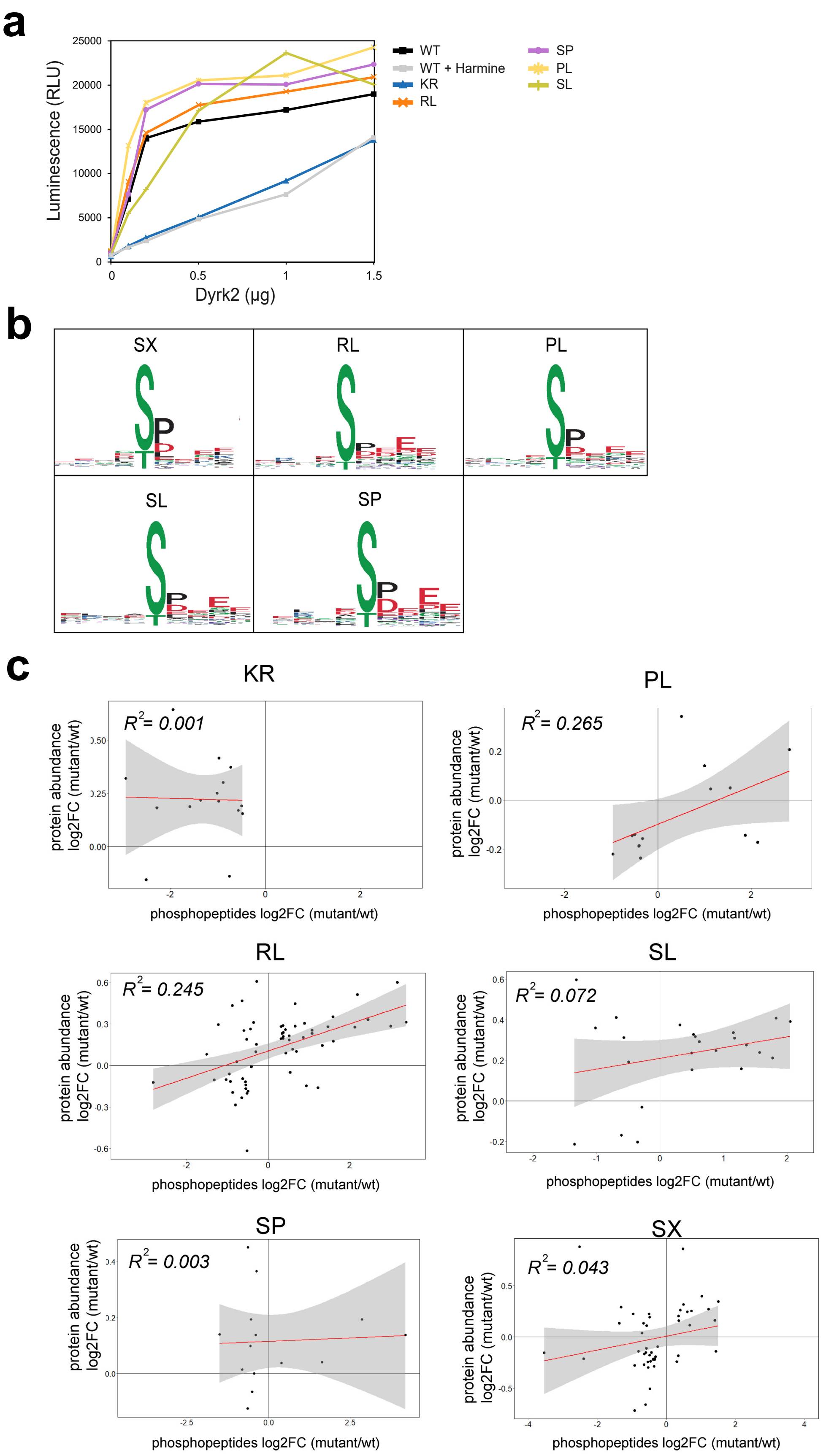

Supplementary Figure 6. Extended phosphoproteomic analysis of cancer-related Dyrk2 mutants. (**a**) *In vitro* kinase assay with Dyrk2 variants purified from SF9 insect cells. The graph shows the Dyrk2 protein amount dependent increase of ATP/ADP conversion indicated by the relative luminescence signal. As control the reaction with Dyrk2 wt was performed in presence of 5 µM harmine (Dyrk2 kinase inhibitor). The values represent the mean of repetitive measurements. (**b**) Phosphomotif analysis of upregulated phosphopeptides in the different Dyrk2 mutant cell lines (log2FC > 0.5, adj. p-value ≤ 0.05). (**c**) Correlation of log2 fold changes between significantly regulated phosphopeptides and the corresponding protein identified in Dyrk2 mutant cells (adj. p-value ≤ 0.05). The statistical analysis and p-value calculation (adj. p-value (FDR)) was performed within the mapDIA package. Source data are provided as source data file.

Supplementary Figure 7

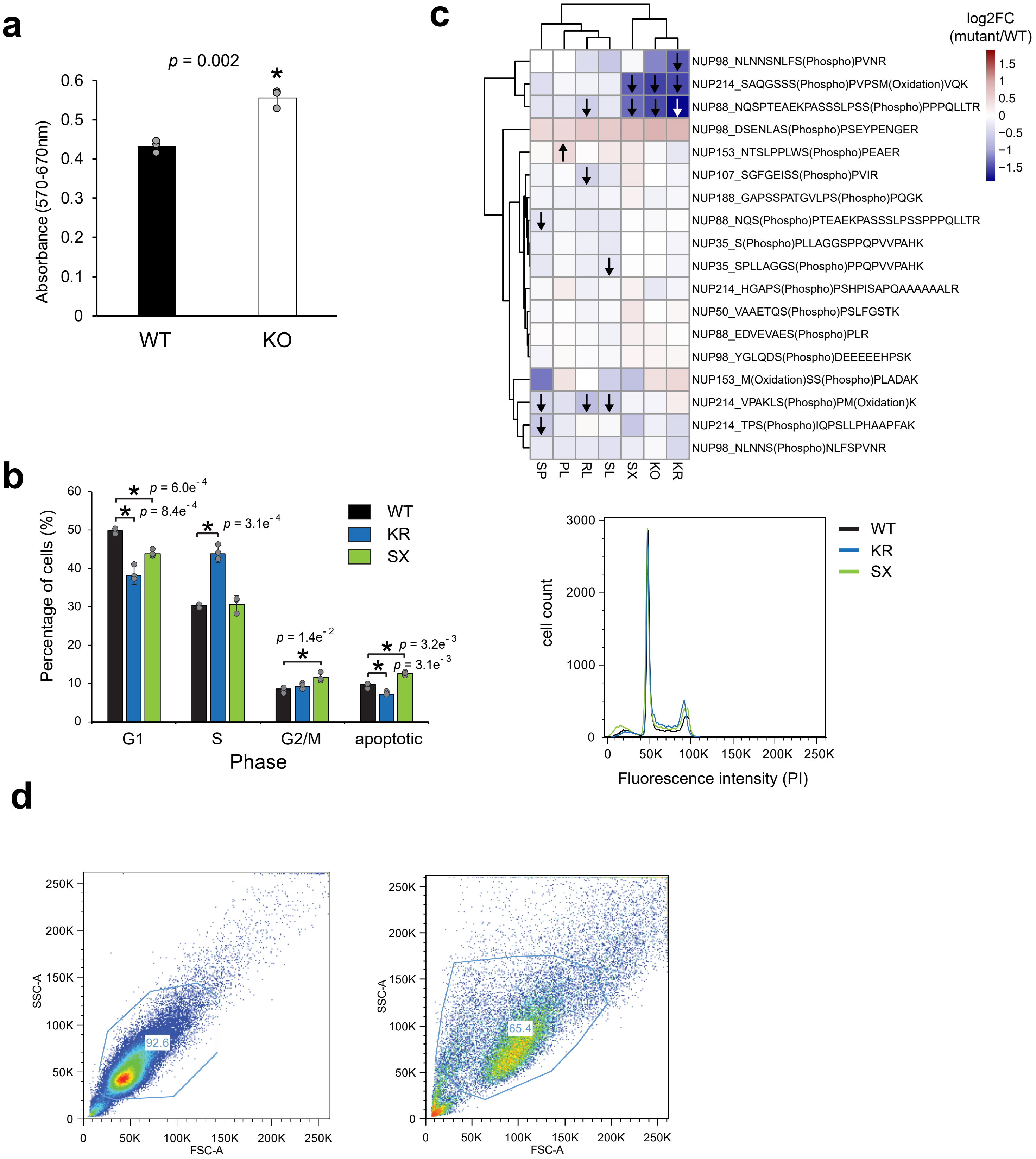

Supplementary Figure 7. Effect of cancer related Dyrk2 mutations on cell proliferation and phosphorylation of nuclear pore subunits (NUPs). (**a**) MTT proliferation assay of CRISPR/Cas9 engineered Dyrk2 KO T-REx HeLa cells. (**b**) Cell cycle analysis of T-REx HeLa cells overexpressing Dyrk2 wt or Dyrk2 KR and Dyrk2 SX, respectively. Overlay of the cell cycle profiles of cells expressing the different Dyrk2 variants measured by flow cytometry (right panel). (**c**) Heatmap showing differences in the abundance of phosphopeptides of nuclear pore components in cells expressing cancer-related Dyrk2 point mutants. The arrow indicates the direction of a significant change in phosphopeptide abundance (adj. p ≤ 0.05). The statistical analysis and p-value calculation (adj. p-value (FDR)) was performed within the mapDIA package. (**d**) Exemplified flow cytometry gating strategy for cell cycle analysis assay (left panel) and Annexin V-FITC apoptosis assay (right panel). Error bars denote the mean value with 95% confidence interval (n = 3 biologically independent experiments). Statistical analysis was performed with two-tailed unpaired Student's t test. The asterisk indicates a significant statistical difference (p-value ≤ 0.05). Source data are provided as source data file.

Supplementary Table 1. List of phosphopeptides containing the Dyrk2 target phosphorylation motif (R/Kxx(x)S/TP).

Protein	phosphosite	KO_log2FC	KR_log2FC	PL_log2FC	RL_log2FC	SL_log2FC	SP_log2FC	SX_log2FC
SQSTM	RSRLT(Phospho)PVS(Phospho)PESSSTEEK	**-2.20**	**-2.22**	-0.27	-0.38	-0.13	-0.24	**-2.21**
MISP	RAST(Phospho)PDWVSEGPQPGLR	**-2.17**	**-2.13**	-0.14	**-0.41**	0.09	**-0.45**	**-2.05**
MEP50	RKET(Phospho)PPPLVPPAAR	**-2.14**	**-2.10**	-0.08	0.20	0.07	-0.18	**-1.97**
PKP4	RSAVS(Phospho)PDLHITPIYEGR	**-2.11**	**-3.30**	-0.04	-0.58	0.01	-0.20	**-1.51**
LIMC1	RSRQT(Phospho)PS(Phospho)PDVVLR	**-2.03**	**-1.80**	0.04	0.10	0.17	-0.28	**-1.96**
SQSTM	RSRLT(Phospho)PVS(Phospho)PESSSTEEK	**-2.01**	**-1.85**	-0.12	-0.20	0.00	0.09	**-1.88**
BET1L	RAQS(Phospho)PGAVEEILDRENK	**-1.98**	**-1.88**	-0.03	-0.07	-0.15	-0.16	**-1.74**
SCRIB	RLRVQS(Phospho)PEPPAPER	**-1.95**	**-1.77**	**-0.41**	-0.24	**-0.32**	**-0.39**	**-1.98**
WDR44	RHLT(Phospho)PEPDIVASTK	**-1.82**	**-1.78**	-0.14	0.04	-0.07	-0.10	**-2.26**
ADDG	RNMS(Phospho)PDLR	**-1.71**	-1.00	0.00	-0.09	-0.07	0.02	**-0.93**
NSF1C	RKKS(Phospho)PNELVDDLFK	**-1.52**	**-2.85**	0.10	0.35	0.24	**0.46**	**-1.32**
LARP1	RAVT(Phospho)PVPTKTEEVSNLK	**-1.48**	**-1.01**	-0.20	-0.10	-0.11	-0.05	**-1.47**
SCRIB	RALS(Phospho)PAELR	**-1.47**	**-1.36**	-0.21	-0.29	-0.15	-0.15	**-1.32**
PTSS2	DAGGPRPES(Phospho)PVPAGR	**-1.38**	**-0.73**	-0.01	-0.37	-0.24	-0.05	**-1.32**
MOCOS	RS(Phospho)LS(Phospho)PQEDALTGSR	**-1.26**	**-1.16**	-0.15	0.31	-0.05	-0.34	**-1.27**
IF4G2	RTQT(Phospho)PPLGQTPQLGLK	**-1.25**	**-1.37**	**-0.42**	-0.22	-0.10	**-0.49**	**-1.40**
ZDHC5	RGVGS(Phospho)PEPGPTAPYLGR	**-1.22**	**-0.98**	-0.31	-0.05	-0.21	**-0.36**	**-1.11**
K1522	LQLERPVS(Phospho)PETQADLQR	**-1.08**	**-1.06**	-0.11	0.05	-0.15	-0.17	**-2.09**
ROA1	KSES(Phospho)PKEPEQLRK	**-1.07**	0.16	0.18	**-1.03**	0.06	0.04	**-0.96**
ZCCHV	KTVFS(Phospho)PTLPAAR	**-1.06**	**-0.81**	-0.27	-0.27	-0.24	-0.29	**-0.97**
BIN1	KGNKS(Phospho)PSPPDGSPAATPEIR	**-1.06**	**-0.79**	**-0.33**	0.01	-0.10	-0.11	**-1.13**

Phosphopeptides were identified with log2FC ≤ -1 in Dyrk2 KO cells. Significant fold changes (adj. p-value ≤ 0.05) are highlighted in bold fonts. Up- or downregulation is shown in red (upregulation) or blue (downregulation) colors, respectively. The statistical analysis and p-value calculation (adj. p-value (FDR)) was performed within the mapDIA package.

Supplementary Table 2. List of identified phosphosites that are reported to be mutated in cancer.

Protein	Position	KO	KR	PL	RL	SL	SP	SX	Reference
ARI1A	S696	0.01	0.06	0.03	-0.02	-0.08	-0.17	0.20	[1, 2]
BRD4	S470	0.63	**-1.57**	**-1.69**	-1.22	**-1.97**	**-1.59**	-0.29	COSU540
NPM	S125	0.32	-0.80	**2.21**	**2.04**	1.20	**2.58**	1.13	[3]
NPM	S260	-0.26	0.22	**-0.57**	**-0.65**	-0.38	**-0.63**	-0.31	[4]
FLNA	S2152	-0.41	-0.37	-0.46	0.07	-0.01	**-1.14**	-0.34	COSU414
RBM10	S797	-0.27	-0.28	-0.17	-0.79	-0.11	-0.41	0.07	[3]
PB1	S636	0.00	0.23	0.13	0.04	-0.03	-0.25	0.34	COSU416
BCLF1	T402	-0.57	0.23	-0.57	0.32	0.62	-0.77	-0.55	[5]
BCLF1	T405	0.06	0.21	-0.13	-0.19	**-0.31**	-0.15	-0.02	[6]

Numbers indicate the measured log2FC in the respective mutant cell lines. Significant fold changes (adj. p-value ≤ 0.05) are highlighted in bold fonts. The statistical analysis and p-value calculation (adj. p-value (FDR)) was performed within the mapDIA package.

Supplementary Table 3. List of Dyrk2 intra cross-links identified in experiments with recombinant Dyrk2 and Dyrk2 expressed in HEK293 cells.

cross-linked peptide	ld score (recomb. Dyrk2)	ld score (AP-XL-MS)
**IIHCDLKPENILLKQQGR-SGIKVIDFGSSCYEHQR**	49.38	43.88
KFAHSILQCLDALHK-TVLPKLVS	25.86	26.17
**EWGNALKGCDDPLFLDFLK-GKLRGPPESR**	40.47	32.64
KPSAAAPAAYPTGR-LLDASKR	37.46	35.99
TVLTTQPNGLTTVGKTGLPVVPER-KPSAAAPAAYPTGR	31.74	26.42
**IIHCDLKPENILLKQQGR-KFAHSILQCLDALHK**	34.9	29.38
KPSAAAPAAYPTGR-GKLRGPPESR	25.13	25.68
KPSAAAPAAYPTGR-TVLPKLVS	32.54	29.87

The table shows all cross-linked peptides that have been identified with ld-score ≥ 25 in both recombinant Dyrk2 purified from SF9 insect cells and Strep/HA-tagged Dyrk2 purified from HEK293 cells. Cross-linked peptides marked in green are confirmed by the known crystal structure of Dyrk2 (PDB: 3K2L).

Supplementary Table 4. List of Dyrk2 mutations annotated in the COSMIC database with the respective number of observation and tissue origin.

AA Mutation	mutation count	Primary Tissue	Pubmed Id	CGP Study
S7L	1	Skin	25303977	-
A13S	1	Large intestine	25344691	-
P15T	1	Liver	-	381
M74I	1	Oesophagus	25839328	-
H79Y	1	Stomach	-	541
G86R	1	Breast	-	414
Q91E	1	Breast	-	414
E95G	1	Large intestine	25344691	-
V103E	1	Thyroid	-	589
T117R	1	Skin	-	540
S139F	1	Prostate	22610119	-
M143V	1	Lung	-	418
G145V	1	Lung	-	417
Q165R	1	Liver	-	322
A188V	1	Large intestine	-	376
R191C	1	Stomach	-	541
**P198L**	1	Central nervous system	16618716	7; 34
D216V	1	Lung	-	418
R221S	1	Lung	-	418
D241N	1	Stomach	-	541
R261Q	1	Stomach	-	541
R268Q	1	Large intestine	25344691	-
R274Q	1	Endometrium	-	419
R294H	1	Endometrium	-	419
C298R	1	Large intestine	24755471	-
M299I	1	Skin	-	540
S305R	1	Large intestine	25344691	-
N307K	1	Kidney	23797736	-
S372R	1	Large intestine	-	376
E375G	2	Large intestine	24755471	-
H376P	1	Lung	-	583
R378C	1	Large intestine	25344691	-
**R378L**	1	Breast	-	414
R378H	1	Large intestine	-	376
T381M	1	Endometrium	-	419
R386C	1	Ovary	-	331
G396R	1	Lung	22980975	-
G396E	1	Liver	-	628
A397S	1	Ovary	21720365	331
A397D	1	Endometrium	-	419
A397V	1	Large intestine	-	376
S407C	1	Endometrium	-	419
T417K	1	Central nervous system	23917401	-
T417M	1	Stomach	-	541
P423S	1	Skin	25303977	-
R451*	1	Stomach	-	541
N454H	1	Endometrium	-	419
K459N	1	Large intestine	24755471	-
Y461C	1	Large intestine	24755471	-
R463H	1	Stomach	-	541
**S471P**	2	Large intestine	-	376
**S471***	1	Breast	15908952	21; 34
**S471L**	1	Breast	22722201	385
N478S	1	Lung	22980975	-
G480R	1	Liver	-	322
S482F	1	Skin	-	540
G489S	1	Liver	-	322
G489D	1	Endometrium	-	419
P506L	1	Skin	-	540
L507P	1	Breast	-	414
F511L	1	Large intestine	-	375
R523H	2	Urinary tract, Kidney	-	413; 416
G527C	1	Skin	25303977	-
R531Q	1	Breast	-	414
H532Y	1	Urinary tract	-	413
R538W	1	Lung	22980975	-
T544I	1	Lung	23033341	-
T557P	1	Kidney	-	416
G558C	2	Kidney, Large intestine	25344691	416
S572P	1	Skin	-	540
L576P	1	Large intestine	25344691	-
A581V	1	Large intestine	25344691	-
M583I	1	Large intestine	25344691	-
S601C	1	Lung	22980975	-

Dyrk2 mutations selected for this study are marked in red and an asterisk indicates stop mutations. The information was downloaded from the COSMIC database (11.08.2015).

Supplementary Table 5. List of oligonucleotides used in this study.

Primer	Sequence
MM003_Dyrk2_seq_fw	CATCCATATGCTGGAG
MM004_Dyrk2_seq_rv	CAGAATCGAGTGGGC
MM005_Dyrk2_R378L_fw	TTGTTACGAGCATCAGCTTGTCTACACGTACATCC
MM006_Dyrk2_R378L_rv	GGATGTACGTGTAGACAAGCTGATGCTCGTAACAA
MM007_Dyrk2_K251R_fw	CAGCACGTGGCCCTA AGGATG GTGCGGAAT G
MM008_Dyrk2_K251R_rv	CATTCCGCACCATCCTTAGGGCCACGTGCT G
MM009_Dyrk2_S471X_fw	ACTGCACTGTCACGACTCTCTGAGATGGCTCTG
MM010_Dyrk2_S471X_rv	CAGAGCCATCTCAGAGAGTCGTGACAGTGCAGT
MM011_Dyrk2_S471P_fw	ACTGTCACGACTCTCCCAGATGGCTCTGTGG
MM012_Dyrk2_S471P_rv	CCACA AGCCATCTGGGAGAGTCGTGACAGT
MM013_Dyrk2_S471L_fw	ACTGCACTGTCACGACTCTCTTAGATGGCTCTG
MM014_Dyrk2_S471L_rv	CAGAGCCATCTAAGAGAGTCGTGACAGTGCAGT
MM113_FLAG_Dyrk2_MultiBac_fw	GGGGACAAGTTTGTACAAAAAAGCAGGCTTC<br>GCGGCCGCATGGACTACAAAGACGATGACGACAAGGGTG<br>GAGGTATGTTAACCAGGAAACCTTCGGCCGCCG
MM115_FLAG_Dyrk2_MultiBac_rv	GGGGACCACTTTGTACAAGAAAGCTGGGTC<br>AAGCTTTTAGCTAACAAGTTTTGGCAACACTG
MM051_Dyrk2_gRNA_1_fw	CACCGACTGACGTTTTCTCCCCGG T
MM052_Dyrk2_gRNA_1_rv	AAACACCGGGGAGAAAACGTCAGTC
MM063_Dyrk2_gRNA_2_fw	CACCGGGACAGCATTCATAGACGGC
MM064_Dyrk2_gRNA_2_frv	AAACGCCGTCTATGAATGCTGTCCC

Supplementary Notes

Supplementary Note 1: Phosphoproteomic analysis of cell lines expressing Dyrk2 mutants by SWATH-MS.

Phosphopeptides were consistently and reproducibly quantified in triplicates with a replicate CV < 16% (**Supplementary Figure 5d – 5f**). We quantified 2888 unique phosphopeptides (peptide FDR ≤ 1%) across the different conditions, and for 2040 phosphosites the localization of the phosphate group within the phosphopeptide could be determined with a false localization rate (FLR) < 1% (localized phosphopeptides). In total we detected 214 differentially abundant phosphopeptides (FLR < 1%, adj. p-value ≤ 0.05, │log2FC│> 1), which represents ~10% of the measured phosphoproteome (**Figure 6b**). Hence, this indicates that changes within the Dyrk2 module are likely to have a broad effect on the cellular phosphoproteome; both through direct phosphorylation and through downstream signal propagation.

Supplementary Note 2: Generation of cancer driver network perturbed by Dyrk2 mutations.

In order to build the network we (i) selected significantly regulated proteins in our PPI and phosphoproteomic data that also are present in the Cancer Census (https://cancer.sanger.ac.uk/census, download 20190410); (ii) mapped the known interactions among the selected proteins based on the PPI repository IID (http://iid.ophid.utoronto.ca/; only experimentally validated interactions); (iii) performed a gene ontology analysis using DAVID (Biological Process; https://david.ncifcrf.gov/), merged significant terms (p-values <0.05) in more general/related terms, and grouped proteins in the graph accordingly (Materials and Methods).

Supplementary Note 3: Effect of Dyrk2 mutations on cellular apoptosis.

Dyrk2 KR and Dyrk2 SX affect the binding to and the phosphorylation of CDPs such as TP53 and CXCR4 that are involved in the regulation of apoptosis [7]. Accordingly, the overexpression of Dyrk2 KR leads to a reduced fraction of apoptotic cells as shown by Annexin-5 apoptosis assay in MDA-MB-231 cells as well as cell cycle analysis in T-REx HeLa cells (**Figure 8c** and **Supplementary Figure 7b**) in keeping with the known pro-apoptotic function of Dyrk2 by phosphorylating TP53 during DNA damage [8]. Although our data strongly indicate that Dyrk2 SX is catalytically inactive its expression results in the opposite effect and increases apoptosis (**Figure 8c** and **Supplementary Figure 7b**).

Compared to Dyrk2 wt or other mutants Dyrk2 SX shows clearly elevated protein levels (**Supplementary Figure 5c**) which may negatively influence cell viability.

Supplementary Note 4: The phosphorylation of specific nuclear pore subunits is affected by Dyrk2 mutations.

Of particular interest in this subnetwork are the CDPs NUP214 and NUP98 whose phosphorylation was downregulated in Dyrk2 KO (NUP214 S657, log2FC = -1.55, adj. p-value = 6.35E-9), KR (NUP214 S657, log2FC = -1.51, adj. p-value = 3.79E-9; NUP98 S612, log2FC = -1.42, adj. p-value = 1.61E-2) and SX (NUP214 S657, log2FC = -1.39, adj. p-value = 4.14E-13) (**Figure 8a**). Notably, NUP98 phosphorylation was also found to be affected in Dyrk2 KO cells but was not considered as significant given the set p-value criteria (NUP98 S612, log2FC = -1.19, adj. p-value = 6.00E-2). Beside NUP214 and NUP98, the phosphorylation of NUP88 (S50), which is not annotated as CDP but reported to be dysregulated in various cancer types [9, 10], was also reduced by both Dyrk2 KO (log2FC = -1.51, adj. p-value = 1.32E-15) and KR (log2FC = -1.86, adj. p-value = 3.13E-12) as well as Dyrk2 SX (log2FC = -1.32, adj. p-value = 4.14E-13).

Supplementary Discussion

Putative functional role of Dyrk2 at the nuclear pore complex.

Intriguingly, the phosphorylation status of certain NUPs such as NUP98 and NUP53 has been shown to be important for the mitotic disassembly of the nuclear pore complex (NPC) [11]. Recently, PLK1 and the CMGC kinase CDK1 were identified to mediate the hyperphosphorylation of these NUPs during mitosis which induces their release from other nuclear pore components ultimately resulting in the disassembly of the NPC. The association of Dyrk2 with the nuclear Y-complex and its direct involvement in the phosphorylation of nuclear pore components suggests a novel functional role of Dyrk2 at the NPC, potentially in the mitotic hyperphosphorylation and disassembly of the NPC, which is affected by certain cancer-related mutations of the kinase.

Future development and improvements of a proteomic workflow for the systematic analysis of disease mutations.

We envisage three main areas of improvements to this pipeline. First, scalability should be improved by (i) using more efficient CRISPR-Cas9 platform to multiplex mutation generation [12]; (ii) automatizing, scaling down and increasing the throughput of AP-MS and BioID-MS experiments [13, 14]; and (iii) performing cross-linking MS on pull-downs [15] and therefore circumventing cumbersome purification procedures. It needs to be noted that not all layers of analyses will be necessary, and will have to be prioritized based on their predictive value. In this context, we found that in contrast to the prevalent use of protein abundance as the key readout for biomarker studies, protein level measurements has proven in our study the least informative. Instead, interactions studies by BioID-MS and phosphoproteome analysis turned out to provide most valuable information about cellular consequences of kinase mutations. Second, we need to define formal scores and visualization methods quantifying perturbations on different cellular layers and the relationship among them that are complementary to conventional damage probability scores. Finally, the technique utilized here should be integrated with orthogonal, high-throughput techniques, that can shed light on additional aspects of proteome organization, such as protein complex profiling by size exclusion chromatography and limited proteolysis [16, 17].

Supplementary References

1. Shain, A.H. *et al.* Exome sequencing of desmoplastic melanoma identifies recurrent NFKBIE promoter mutations and diverse activating mutations in the MAPK pathway. *Nat Genet* **47**, 1194-1199 (2015).
2. Chou, W.C. *et al.* Genes involved in angiogenesis and mTOR pathways are frequently mutated in Asian patients with pancreatic neuroendocrine tumors. *Int J Biol Sci* **12**, 1523-1532 (2016).
3. Zehir, A. *et al.* Mutational landscape of metastatic cancer revealed from prospective clinical sequencing of 10,000 patients. *Nat Med* **23**, 703-713 (2017).
4. Kumar, A. *et al.* Substantial interindividual and limited intraindividual genomic diversity among tumors from men with metastatic prostate cancer. *Nat Med* **22**, 369-378 (2016).
5. Bruno, A. *et al.* Mutational analysis of primary central nervous system lymphoma. *Oncotarget* **5**, 5065-5075 (2014).
6. Shi, J. *et al.* Somatic Genomics and Clinical Features of Lung Adenocarcinoma: A Retrospective Study. *PLoS Med* **13**, e1002162 (2016).
7. Kremer, K.N. *et al.* CXCR4 chemokine receptor signaling induces apoptosis in acute myeloid leukemia cells via regulation of the Bcl-2 family members Bcl-XL, Noxa, and Bak. *J Biol Chem* **288**, 22899-22914 (2013).
8. Taira, N., Nihira, K., Yamaguchi, T., Miki, Y. & Yoshida, K. DYRK2 is targeted to the nucleus and controls p53 via Ser46 phosphorylation in the apoptotic response to DNA damage. *Mol Cell* **25**, 725-738 (2007).
9. Agudo, D. *et al.* Nup88 mRNA overexpression is associated with high aggressiveness of breast cancer. *Int J Cancer* **109**, 717-720 (2004).
10. Zhang, Z.Y. *et al.* Nup88 expression in normal mucosa, adenoma, primary adenocarcinoma and lymph node metastasis in the colorectum. *Tumour Biol* **28**, 93-99 (2007).
11. Linder, M.I. *et al.* Mitotic Disassembly of Nuclear Pore Complexes Involves CDK1- and PLK1-Mediated Phosphorylation of Key Interconnecting Nucleoporins. *Dev Cell* **43**, 141-156 e147 (2017).
12. Kurata, M. *et al.* Highly multiplexed genome engineering using CRISPR/Cas9 gRNA arrays. *PLoS One* **13**, e0198714 (2018).
13. Hein, M.Y. *et al.* A human interactome in three quantitative dimensions organized by stoichiometries and abundances. *Cell* **163**, 712-723 (2015).
14. Huttlin, E.L. *et al.* Architecture of the human interactome defines protein communities and disease networks. *Nature* **545**, 505-509 (2017).
15. Makowski, M.M., Willems, E., Jansen, P.W. & Vermeulen, M. Cross-linking immunoprecipitation-MS (xIP-MS): Topological Analysis of Chromatin-associated Protein Complexes Using Single Affinity Purification. *Mol Cell Proteomics* **15**, 854-865 (2016).
16. Heusel, M. *et al.* Complex-centric proteome profiling by SEC-SWATH-MS. *Mol Syst Biol* **15**, e8438 (2019).
17. Feng, Y. *et al.* Global analysis of protein structural changes in complex proteomes. *Nat Biotechnol* **32**, 1036-1044 (2014).
