# Supplementary material for: Multi-layered proteomic analyses decode compositional and functional effects of cancer mutations on kinase complexes
Source: Nat Commun. 2020 Jul 16;11:3563. doi: 10.1038/s41467-020-17387-y (PMC7366679; doi:10.1038/s41467-020-17387-y)
Supplement: Supplementary file 3 — Reporting Summary [file 41467_2020_17387_MOESM3_ESM.pdf]

nature research

Corresponding author(s): Ruedi Aebersold, Matthias Gstaiger, Martin Mehnert

Last updated by author(s): Jun 19, 2020

Reporting Summary

Nature Research wishes to improve the reproducibility of the work that we publish. This form provides structure for consistency and transparency in reporting. For further information on Nature Research policies, see our Editorial Policies and the Editorial Policy Checklist.

Statistics

For all statistical analyses, confirm that the following items are present in the figure legend, table legend, main text, or Methods section.

n/a	Confirmed	
☐	☒	The exact sample size (n) for each experimental group/condition, given as a discrete number and unit of measurement
☐	☒	A statement on whether measurements were taken from distinct samples or whether the same sample was measured repeatedly
☐	☒	The statistical test(s) used AND whether they are one- or two-sided<br>*Only common tests should be described solely by name; describe more complex techniques in the Methods section.*
☒	☐	A description of all covariates tested
☒	☐	A description of any assumptions or corrections, such as tests of normality and adjustment for multiple comparisons
☐	☒	A full description of the statistical parameters including central tendency (e.g. means) or other basic estimates (e.g. regression coefficient) AND variation (e.g. standard deviation) or associated estimates of uncertainty (e.g. confidence intervals)
☐	☒	For null hypothesis testing, the test statistic (e.g. F, t, r) with confidence intervals, effect sizes, degrees of freedom and P value noted<br>*Give P values as exact values whenever suitable.*
☒	☐	For Bayesian analysis, information on the choice of priors and Markov chain Monte Carlo settings
☒	☐	For hierarchical and complex designs, identification of the appropriate level for tests and full reporting of outcomes
☒	☐	Estimates of effect sizes (e.g. Cohen's d, Pearson's r), indicating how they were calculated

Our web collection on statistics for biologists contains articles on many of the points above.

Software and code

Policy information about availability of computer code

Data collection	Mass spectrometry data were acquired on AB SCIEX mass spectrometers operated with SCIEX Analyst v.1.7 software or on Thermo Fisher Scientific mass spectrometers operated with Xcalibur 4.1 software. For flow cytometry BD LSRFortessa (BD Biosciences) flow cytometer was used. For FACS sorting BD Facs Aria IIIu sorter (BD Biosciences) was used.
Data analysis	Data were analyzed using public software: MaxQuant v1.5.2.8, X!TANDEM Jackhammer (2013.06.15.1), Comet (2016.01 rev.3), MyriMatch v2.1.138, omssacl 946 (version 2.1.9), OpenSWATH v2.1 , ProteoWizard (3.0.8851), PyProphet v2.0, LuciPHOr2, mapDIA v1.2.1, xQuest v2.1.5, Skyline v4.1, R v3.6.1, DAVID Bioinformatics v6.8, Cytoscape (v3.6.0), FlowJo v9.7.6, Optimized CRISPR Design web tool v1.3 (http://crispr.mit.edu), ImageJ FIJI v1, SAINTexpress (https://www.crapome.org/),<br>custom R script code supporting the findings of this study are available from the corresponding author upon request

For manuscripts utilizing custom algorithms or software that are central to the research but not yet described in published literature, software must be made available to editors and reviewers. We strongly encourage code deposition in a community repository (e.g. GitHub). See the Nature Research guidelines for submitting code & software for further information.

Data

Policy information about availability of data

All manuscripts must include a data availability statement. This statement should provide the following information, where applicable:

- Accession codes, unique identifiers, or web links for publicly available datasets
- A list of figures that have associated raw data
- A description of any restrictions on data availability

The mass spectrometry proteomics data have been deposited to the ProteomeXchange Consortium via the PRIDE partner repository with the dataset identifier PXD015687

Field-specific reporting

Please select the one below that is the best fit for your research. If you are not sure, read the appropriate sections before making your selection.

- [x] Life sciences
- [ ] Behavioural & social sciences
- [ ] Ecological, evolutionary & environmental sciences

For a reference copy of the document with all sections, see nature.com/documents/nr-reporting-summary-flat.pdf

Life sciences study design

All studies must disclose on these points even when the disclosure is negative.

Sample size	Statistical methods were not used to predetermine sample size. For each experiment at least n = 3 biologically independent samples were performed in order to allow statistical testing.
Data exclusions	No data were excluded.
Replication	All attempts at replication were successful. Samples were analyzed in triplicates. Reproducibility was determined by Pearson correlation.
Randomization	Samples were randomized for sample preparation in each experiment. Here the samples were not allocated in specific groups, instead the samples were randomized by names at the beginning of the experiment.
Blinding	The investigators were blinded to group allocation during data collection and analysis.

Reporting for specific materials, systems and methods

We require information from authors about some types of materials, experimental systems and methods used in many studies. Here, indicate whether each material, system or method listed is relevant to your study. If you are not sure if a list item applies to your research, read the appropriate section before selecting a response.

Materials & experimental systems

n/a	Involved in the study	
☐	☒	Antibodies
☐	☒	Eukaryotic cell lines
☒	☐	Palaeontology and archaeology
☒	☐	Animals and other organisms
☒	☐	Human research participants
☒	☐	Clinical data
☒	☐	Dual use research of concern

Methods

n/a	Involved in the study	
☒	☐	ChIP-seq
☐	☒	Flow cytometry
☒	☐	MRI-based neuroimaging

Antibodies

Antibodies used	anti-Dyrk2 (HPA027230, Sigma), anti-DDB1 (D4C8) (#6998, Cell Signaling), anti-Ubr5 (D6O8Z) (#65344, Cell Signaling), anti-VprBP (D5K5V) (#14966, Cell Signaling), anti-FLAG (F3165, Sigma), anti-HA (HA.11,901513, BioLegend), anti-Actin (ab179467, Abcam)
Validation	anti-Dyrk2: https://www.sigmaaldrich.com/catalog/product/sigma/hpa027230?lang=de&region=CH<br>anti-DDB1: https://www.cellsignal.com/products/primary-antibodies/ddb-1-d4c8-rabbit-mab/6998<br>anti-Ubr5: https://www.cellsignal.com/products/primary-antibodies/ubr5-d6o8z-rabbit-mab/65344<br>anti-FLAG: https://www.sigmaaldrich.com/catalog/product/sigma/f3165?lang=de&region=CH<br>anti-HA: https://www.biolegend.com/en-us/products/anti-ha-11-epitope-tag-antibody-11071<br>anti-Actin: https://www.abcam.com/actin-antibody-epr16769-ab179467.html<br>anti-VprBP: https://www.cellsignal.com/products/primary-antibodies/vprbp-d5k5v-rabbit-mab/14966

Eukaryotic cell lines

Policy information about cell lines

Cell line source(s)	HeLa Flp-In (R71407) and HEK293 Flp-In cells (R78007) were purchased from Invitrogen, MDA-MB-231 (cells were purchased from ATCC, SF9 cells were purchased from Invitrogen (#11496015)
Authentication	Cell authentication was performed by SNV profiles.
Mycoplasma contamination	Before performing the study all cell lines were tested and confirmed to be negative for mycoplasma contamination.

Commonly misidentified lines (See ICLAC register)	No commonly misidentified cell lines were used.

Flow Cytometry

Plots

Confirm that:

- [x] The axis labels state the marker and fluorochrome used (e.g. CD4-FITC).
- [x] The axis scales are clearly visible. Include numbers along axes only for bottom left plot of group (a 'group' is an analysis of identical markers).
- [x] All plots are contour plots with outliers or pseudocolor plots.
- [x] A numerical value for number of cells or percentage (with statistics) is provided.

Methodology

Sample preparation	Cells were washed with 1xPBS, cell cycle assay: incubation with Propidium Iodide and RNase for 30 min at 37°C,<br>Annexin V apoptosis assay: cells were resuspended in Annexin binding buffer, then Annexin V-FITC and Propidium Iodide was added to cells immediately before flow cytometry analysis
Instrument	BD LSRFortessa (BD Biosciences)
Software	FlowJo v9.7.6
Cell population abundance	no sorting was performed
Gating strategy	cell cycle analysis: first gate was set on intact cells (SSC-A/FSC-A) , cell debris was excluded, second gate was set on singlets<br>Annexin V-FITC apoptosis assay: gate was set on intact cells (SSC-A/FSC-A), cell debris was excluded

- [x] Tick this box to confirm that a figure exemplifying the gating strategy is provided in the Supplementary Information.
